# CRISPR/Cas9 Induced Somatic Recombination at the *CRTISO* Locus in Tomato

**DOI:** 10.3390/genes12010059

**Published:** 2020-12-31

**Authors:** Ilan Ben Shlush, Aviva Samach, Cathy Melamed-Bessudo, Daniela Ben-Tov, Tal Dahan-Meir, Shdema Filler-Hayut, Avraham A. Levy

**Affiliations:** Department of Plant and Environmental Sciences, The Weizmann Institute of Science, Rehovot 7610001, Israel; ilansimp@gmail.com (I.B.S.); aviva.samach@weizmann.ac.il (A.S.); Cathy.Bessudo@weizmann.ac.il (C.M.-B.); daniela.ben-tov@weizmann.ac.il (D.B.-T.); tal.dahan@weizmann.ac.il (T.D.-M.); shdema.filler@weizmann.ac.il (S.F.-H.)

**Keywords:** DNA double-strand break repair, homologous recombination, CRISPR/Cas9, inter homologous somatic recombination, targeted gene conversion, targeted crossover, tomato

## Abstract

Homologous recombination (HR) in somatic cells is not as well understood as meiotic recombination and is thought to be rare. In a previous study, we showed that Inter-Homologous Somatic Recombination (IHSR) can be achieved by targeted induction of DNA double-strand breaks (DSBs). Here, we designed a novel IHSR assay to investigate this phenomenon in greater depth. We utilized F_1_ hybrids from divergent parental lines, each with a different mutation at the *Carotenoid isomerase* (*CRTISO*) locus. IHSR events, namely crossover or gene conversion (GC), between the two *CRTISO* mutant alleles (tangerine color) can restore gene activity and be visualized as gain-of-function, wildtype (red) phenotypes. Our results show that out of four intron DSB targets tested, three showed DSB formation, as seen from non-homologous end-joining (NHEJ) footprints, but only one target generated putative IHSR events as seen by red sectors on tangerine fruits. F_2_ seeds were grown to test for germinal transmission of HR events. Two out of five F_1_ plants showing red sectors had their IHSR events germinally transmitted to F_2_, mainly as gene conversion. Six independent recombinant alleles were characterized: three had truncated conversion tracts with an average length of ~1 kb. Two alleles were formed by a crossover as determined by genotyping and characterized by whole genome sequencing. We discuss how IHSR can be used for future research and for the development of novel gene editing and precise breeding tools.

## 1. Introduction

Homologous recombination (HR) is the most common way to generate genetic variation in sexually reproductive organisms. It is achieved through the exchange of parental chromosomal segments between two homologous chromosomes followed by segregation of the new recombinant alleles into separate germinal cells [1]. This process is initiated by Spo11-mediated DNA double-strand breaks (DSBs) happening during meiosis at various locations along the chromosomes [1,2,3,4,5]. The outcomes of HR are crossovers (COs) in case of a reciprocal exchange of homologous segments, or non-crossovers (NCOs) in case of non-reciprocal transfer of a small DNA segment from one parent to the other, which is also known as gene conversion (GC) [6]. The ability to obtain plants with a combination of desired traits during a breeding program is limited by several features of meiotic recombination such as genetic interference, the difficulty to segregate linked favorable and unfavorable traits, non-random Spo-11-induced DSBs along the chromosome [3] and crossover suppression in heterochromatic regions [6]. Moreover, generating genetic variation of new desirable traits in vegetatively propagated crops cannot rely on meiotic recombination.

In plants, germ line cells giving rise to pollen or egg cells are formed late in development, when somatic meristematic cells differentiate into floral meristems [7]. Hence, inducing HR in somatic cells in a precise manner could lead to targeted recombination that can be transmitted to the germinal cells, paving the way to overcome meiotic recombination limitations. This could also generate genetic diversity in vegetatively propagated crops. HR in somatic cells is not as well understood as meiotic recombination and it is thought to be extremely rare, but a few studies showed that it is possible to induce HR by gamma irradiation in *Vicia faba* [8] and by transposon in tobacco (*Nicotiana tabacum*) as shown in the *Hyrec* mutant [9]. In addition, in recent work we showed that Inter-Homologous Somatic Recombination (IHSR) repair is possible in tomato plants (*Solanum lycopersicum*) and can be achieved by targeted induction of DNA double-strand breaks (DSBs) using the CRISPR-Cas9 system [10]. Only a few events were analyzed, showing the occurrence of targeted gene conversion, and one putative CO event was identified. Conversion tracts were interrupted, possibly as a result of mismatch repair. These findings raised a number of questions that require further investigation to better understand the somatic HR repair mechanisms.

In this study, we designed a new assay for tomatoes to induce DSBs in somatic cells using the CRISPR-Cas9 system, targeting introns in the *Carotenoid isomerase* (*CRTISO*) locus, originally referred to as *tangerine* [11], in a way that enables us to promote, screen, and characterize HR repair events based on a gain-of-function color assay in a hybrid between two parents with a sequenced genome. We describe the assay design, and the analysis of DSB repair products, mainly non-homologous end-joining (NHEJ) or repair via HR between homologues resulting in GC or CO.

## 2. Materials and Methods

### 2.1. Plant Material

Micro-Tom (*Solanum lycopersicum*) *tangerine* tomato mutant plants #18-3 described by [12], and M82 *tangerine* e3406 [13], were grown in one-liter pots and five-liter pots respectively in a greenhouse for crossing. The controlled climate conditions were of 26 ± 1 °C and under 12 h light period. The intron targeting cassettes were transformed into F_1_ hybrids. The Cas9-only cassette was transformed into the M82 parental genotype, or into F_1_ hybrids. Following transformation, regeneration, and selections, the plantlets were transferred from rooting media in a tissue culture growing room to five-liter pots. F_2_ and F_3_ progeny plants were sown in five-liter pots.

### 2.2. Plasmids Construction

Single guide RNAs (sgRNAs) targeting four different introns within the *CRTISO* gene between the two *tangerine* mutations were designed and checked for off-targets using Ensembl Plants [14] and cloned into a construct containing Streptococcus pyogenes Cas9 (SpCas9) gene. Cloning was performed by utilizing the Golden Braid (GB) system [15]. The constructs were composed of SpCas9 that was expressed under constitutive Ubiquitin4-2 promoter from *Petroselinum crispum* (PcUbi4-2) and Pea3A terminator [16]. The sgRNA was expressed under the control of *Arabidopsis thaliana* U6-26 RNA Pol III promoter and the Kanamycin resistance gene (Kana) was expressed under the Nopaline Synthase (Nos) promoter and Nos terminator (referred to as Nos:NptII:Nos).

### 2.3. Plant Transformation and Genotyping

Plasmids were transformed into *Agrobacterium tumefaciens* of the strain GV3101 by electroporation. The colonies were grown on LB plates with 200 μg/mL spectinomycin and 50 μg/mL gentamycin antibiotics. F_1_ cotyledons were transformed with the plasmid-carrying *Agrobacterium*. Swollen green cotyledons were transferred through several selection phases. Transformation and selection protocols were previously described [12]. Regenerated plantlets (containing T-DNA with Kanamycin resistance gene) were transferred to rooting media with Kanamycin and IBA (rooting hormone). Two to three small tomato leaflets were collected from different branches into 1.5 mL tubes and were put inside liquid nitrogen. The frozen leaflets were crushed by electric drill with designated frozen plastic sticks and DNA extraction was performed as previously described [12]. Verification of transgenic F_1_ plants was done by PCR amplification of SpCas9 sequence from genomic DNA. Amplification was done by 35 cycles PCR using RedTaq^®^ ReadyMix™ (Sigma-Aldrich, St Louis, MO, USA). Primers used for the amplification are specified in Supplementary Table S1. F_2_ and F_3_ plants were tested as well in a similar fashion. Genomic DNA samples were used for PCR amplification of the *CRTISO* gene and its flanking regions. PCR fragments were amplified by 35 cycles using KAPA HiFi HotStart ReadyMix (KAPA Biosystems-50 μL reactions according to the manufacturer’s protocol), and sequenced by Sanger sequencing. The list of primers is given in Supplementary Table S2.

### 2.4. NHEJ Analysis Using Illumina HTS

Genomic DNA from F_1_ and M82 transgenic plants for all four SpCas9 + gRNA constructs were amplified by 18 cycles of PCR, using 50 μL reaction of KAPA HiFiTM ReadyMix (KAPA-BIOSYSTEMS) to produce amplicons in order to build libraries for high-throughput Illumina^®^ sequencing. Three sets of primers, targeting amplicons of ~300 bp, were designed to amplify two CRISPR target sites at each amplicon. Targets 7–8 and 8–9 were amplified from both sides of the target due to an overlap of two amplicons (Supplementary Tables S3 and S4). Primers were 20–60 bp from each DSB site and were attached at the 5′ end with specific barcodes that had varied length between 4–7 bp with a unique sequence to indicate the plant origin. Deep sequencing was done using Illumina NextSeq 550 system at the Life Sciences Core facilities unit at the Weizmann Institute of Science. The sample sequences were analyzed using NGS Cas-analyzer as described by [17].

### 2.5. HR Events Analysis in the CRTISO Region

The SL4.0 chromosome 10 region between 61,795,407 and 61,787,754 (7653 bp), spanning the *tangerine* mutations of both Micro-Tom and M82 was sequenced for each IHSR candidate plant. We used Sanger sequencing of PCR products in *CRTISO* regions of heterozygous F_2_ plants or homozygous F_3_ plants. For F_2_ plants that did not have seeds, and therefore could not give homozygous F_3_ plants, PCR products were cloned into pGEM T-easy and single colonies were sent for sequencing. Primers used for the amplification are specified in Supplementary Table S5.

### 2.6. CO Events Analysis Using Illumina Whole Genome Sequencing (WGS)

DNA was purified from leaves of F_2_, and F_3_ plants using a DNA purification kit (MACHEREY-NAGEL^®^) and then 300 ng sheared by sonication (Bioruptor ^®^, Diagenode) to 200–500 bp. A total of 10 ng of fragmented DNA per plant was used for libraries preparation as described by [18]. High-throughput sequencing was performed at the Life Sciences Core facilities unit at the Weizmann Institute of Science with the Illumina NovaSeq 6000, 150 bp paired-end reads.

The whole genome sequencing of tomato plants was aligned to the Heinz tomato genome, version SL4.0 (Sol Genomic Network), using BWA [19] and Samtools [20]. Potential PCR duplicates were removed using Samtools rmdup and by the Picard tool FilterSamreads [21]. The reads were viewed and documented using the IGV browser [22]).

## 3. Results

### 3.1. Gain-of-Function Phenotypic Assay Design for HR Events Identification

**Figure 1:**
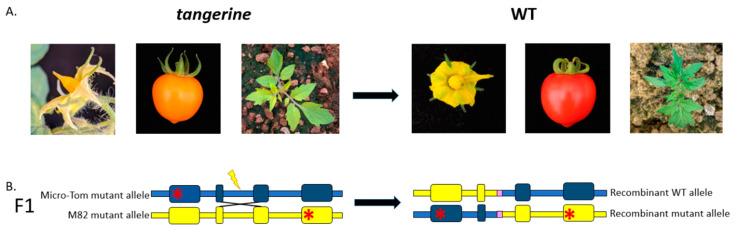
Phenotypic Inter-Homologous Somatic Recombination (IHSR) assay in the *Carotenoid isomerase* (*CRTISO*) gene utilizing visible gain-of-function events in F_1_ plants. (**A**) *CRTISO* recessive mutant *tangerine* phenotypes seen as pale yellow petals, orange fruits, and yellowish young leaves. IHSR can lead to the correction of one mutant allele to the dominant WT allele and hence the plant will show WT phenotypes of yellow petals, red fruit, and green young leaves. (**B**) F_1_ hybrid has two recessive *CRTISO* alleles (schematic not-to-scale representation). Each allele mutation is represented by a red asterisk. Exons are shown as wide boxes. The Micro-Tom background is blue and the M82 background yellow. The lightning bolt corresponds to a DSB site; the NHEJ footprint is in pink; and the X structure shows the site of IHSR repair induced by double-strand break (DSB). IHSR can generate recombinant WT or mutant alleles. NHEJ footprints that would occur after IHSR repair are not expected to change the WT phenotype since the DSB sites are in introns in non-splicing sites.

**Figure 2:**
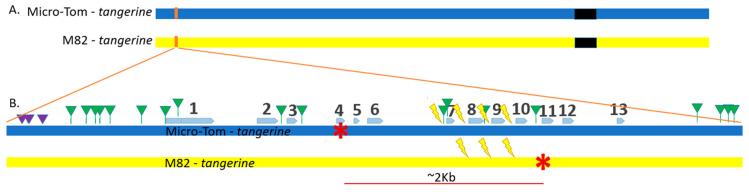
*CRTISO* gene map of targeted DSB IHSR assay. (**A**) Tomato Chromosome#10 scheme. The centromere is shown in black. The *CRTISO* gene is located at the sub-telomeric region of chromosome 10, on the reverse strand. Its approximate location is in orange. (**B**) The scheme of *CRTISO* gene region with exon 1 close to the telomere and exon 13 to the centromere. Exons are marked by blue arrows. Micro-Tom *tangerine* allele and its-A mutation at exon 4, and M82 allele G to A mutation at exon 11 are marked by red asterisks. The distance between the mutations is ~2 kb. Indels are marked by purple triangles. Single nucleotide polymorphisms (SNPs) are marked by green triangles. Four gRNAs for DSBs (lightning bolts) in between the two mutations were designed to target introns, and named according to their location; for example, the target in intron between exon 8 and exon 9 was called target 8–9. Three targets can break both alleles. Target 6–7 is allele specific, targeting only the Micro-Tom allele due to a SNP resulting in an NGG forming a protospacer adjacent motif (PAM).

A recessive mutation in the *Carotenoid isomerase* (*CRTISO*) gene from the carotenoid biosynthesis pathway in tomatoes gives rise to a tangerine phenotype [11], which causes pale yellow petals, orange fruits, and a less robust yet still visible phenotype with yellowish young leaves (Figure 1). We took advantage of the availability of several *CRTISO* mutants to design an assay whereby intergenic HR between mutant alleles can restore the WT yellow petals and red fruit phenotype. DSB induction was targeted to introns so that the recombination product can tolerate NHEJ mutations without affecting WT activity (Figure 1). Two *CRTISO* mutants in the background of divergent parental lines were used: one in Micro-Tom (MT), *tangerine* 18-3, and one in the cultivar M82 background, *tangerine* e3406; the distance between the two mutations is ~2 Kb. Both mutations result in a homozygote recessive tangerine phenotype (see red stars in Figure 2). MT *CRTISO* has a “-A” frameshift mutation in exon 4 that was generated by CRISPR-Cas9 creating a premature stop codon [12], and M82 *CRTISO* has a G to A missense mutation in exon 11, generated by Ethyl Methanesulfonate (EMS) mutagenesis [13]. Crossing these lines resulted in tangerine F_1_ hybrid plants that had one mutant allele from each parent, whose origin could be differentiated by unique insertions or deletions (indels), and single nucleotide polymorphisms (SNPs) (Figure 2B, Supplementary Table S6). Plant and fruit size of the F_1_ hybrid are similar to M82, however, fruit shape is rounded and resembles MT. Four different CRISPR-Cas9 gRNA targets were designed to induce DSBs in introns in between the mutations in F_1_ plants to allow HR to take place in somatic cells following transformation (Figure 2B, Supplementary Tables S3 and S6). In case one mutant allele would be repaired via somatic HR using the homologue as a repair template, a WT allele would be formed, leading to chimeric *CRTISO* plants with whole WT branches of red fruits for early HR events, or red sectors in orange fruits for later somatic events. Target locations were 180 to 279 bp from each other and were chosen based on several criteria: 1. Targeting introns to prevent gene activity disruption by NHEJ footprints, which could be formed after HR. In exons, recurring cuts would affect gene activity and our ability to select IHSR events based on the phenotype. 2. Putative off-targets were checked to avoid collateral genome damage as much as possible. 3. Splicing sites were verified to be distant from the targets to avoid intron splicing interruption (Supplementary Table S3). For the target between exon 6 and 7 (6–7 gRNA), we took advantage of the T to G SNP that results in an NGG protospacer adjacent motif (PAM), hence targeting a DSB only in the MT allele (Figure 2). The distance between the *tangerine* mutations is ~2 kb, making it very unlikely for meiotic recombination to occur in the progeny of F_1_ plants. SpCas9 presence was verified by PCR, using SpCas9 primers (Supplementary Table S1). SpCas9 positive plants were continuously monitored for WT phenotypes, focusing mainly on the flowers and fruits. Total transformation success rates were 76.40% and 65.12% for F_1_ and M82 respectively.

### 3.2. Three out of Four CRTISO Targets Induce Efficient NHEJ DSB Repair

**Figure 3:**
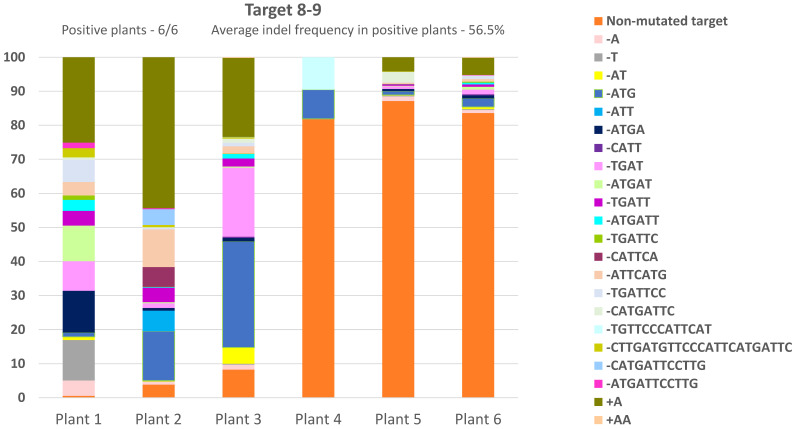
NHEJ frequency in F_1_ plants of *CRTISO* assay DSB target 8–9. Illumina NextSeq high-throughput sequencing results for F_1_ plants analyzed using NGS Cas-Analyzer and presented for *CRTISO* assay DSB target 8–9. Six F_1_ plants were analyzed. The indels frequency was calculated by the number of indel reads out of the total number of reads (including non-mutated target footprint) and shown as a percentage. No indels were detected in three control plants with Cas9 only.

NHEJ DSB repair was evaluated for the four *CRTISO* targets in six F_1_ lines per target. We analyzed Cas9 and gRNA NHEJ footprints using Illumina sequencing of PCR products flanking each gRNA target (Figure 3 and Supplementary Figure S1). F_1_ lines with Cas9 only were tested as controls (Supplementary Table S7). Indels frequency, a traceable outcome of NHEJ DSB repair, was measured per F_1_ line. Three out of the four targets showed significant NHEJ repair activity. Target 6–7, as predicted and designed, showed indels only in the MT allele. Four out of six target 6–7 lines were positive, with average indel frequency of 21.7% in positive plants (Supplementary Figure S1A). Target 7–8 had four out of six positive lines, with average indel frequency of 92.5% in positive plants (Supplementary Figure S1B). All six target 8–9 F_1_ lines were positive, with average indel frequency of 56.5% in positive plants (Figure 3). Target 9–10 did not show any activity (Supplementary Figure S1D).

### 3.3. CRTISO Target 8–9 Gives Germinal Transmission of Somatic DSB IHSR Events

**Figure 4:**
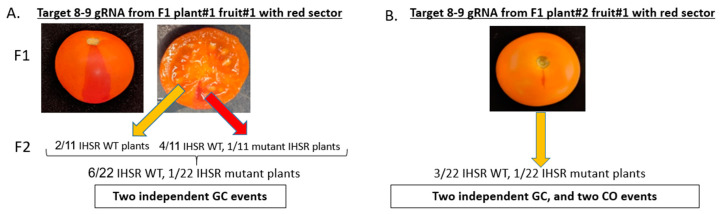
*CRTISO* target 8–9 induces IHSR events. Red sector fruits indicating late IHSR event, were detected in *CRTISO* DSB target 8–9 only. (**A**) The red sector of F_1_ 8–9 DSB target plant#1 fruit#1 reached the mesocarp, unlike all other smaller red sector fruits that remained in the pericarp. Seeds were collected separately from the tangerine and red WT parts of the fruit. Eleven seeds of each tangerine and red WT sector were grown. Four IHSR WT and one IHSR mutant progeny were found from the red WT sector. Two more IHSR WT progeny were found from the tangerine fruit part, leading to a total of seven IHSR progeny out of 22 F_2_ plants. (**B**) The red sector of F_1_ 8–9 DSB target plant#2 fruit#1 was present in the pericarp only, therefore, seeds were collected as one pool. Twenty-two seeds were grown. Three IHSR WT and one IHSR double mutant progeny were found out of 22 F_2_ plants.

Monitoring F_1_ plants phenotypes did not result in any WT flowers expected for IHSR events. However, at later growth stages as the fruits ripened, we found a few plants with red sectors on orange (tangerine) fruits, a sign of late IHSR events (Figure 4). All the plants that had fruits with a red sector were from target 8–9. Five out of eighteen Cas9- independent transgenic plants from this target had fruits with red sectors (Supplementary Table S7). Overall, we obtained ten fruits with red sectors. Two out of the five transgenic plants had three fruits with red sectors, one plant had two fruits with red sectors, and two plants had just one fruit with red sectors. We noticed that these fruits were located on different branches, which may indicate independent IHSR events. Each fruit seed was separately collected for subsequent monitoring. F_1_ plants of targets 6–7, 7–8, and 9–10 did not show any WT phenotype sectors (Supplementary Table S7). As expected, all M82 control target plants and Cas9-only cassette plants (of both M82 and F_1_) displayed the tangerine phenotype with no red sectors. Plant#1 and plant#2 of target 8–9, each containing three fruits with red sectors, were from lines that showed 99.5% and 96.3% indel frequencies respectively (Figure 3C). Both plant#1 and plant#2 of target 8–9 had one red sector fruit, with germinally transmitted IHSR (Figure 4A,B). Plant#5 of target 8–9, which had one red sector fruit, showed only 14.5% indel frequency (Figure 3C). Obtaining detectable somatic recombination events in target 8–9 only raises questions as to the genomic context necessary of targeted IHSR to occur.

### 3.4. CRTISO Target 8–9 Activity in Somatic Tissue Leads to Germinal Transmission of GC and CO Events

To test the germinal transmission and to conduct molecular analysis of IHSR events, each red sector fruit from target 8–9 was collected and its seeds were extracted separately. Three orange tangerine fruits and their seeds were collected from each F_1_ plant and from five plants of the other targets and controls. Seeds were sown to compare the progeny phenotype of the orange fruits to the progeny of the fruits with the red sector. In the tangerine fruits progeny, neither red sector fruits nor WT fruits were detected, indicating no meiotic activity of SpCas resulting in visible IHSR events. We hypothesized that seeds extracted from fruits with red sectors might give rise to either WT or tangerine F_2_ plants, depending on whether the somatic cells that underwent IHSR (red sector) differentiate into germline cells producing gametes. Fruit#1 from F_1_ plant#1 had a large red sector (Figure 4A) compared to the other fruits which had a rather small red sector (Figure 4B). The large red sector reached the mesocarp (Figure 4A), unlike the small sectors which were limited to the pericarp. The large red sector (Figure 4A) was carefully cut out and the seeds were collected separately from the seeds of the orange part of the fruit. To test whether only the red sector seeds would give rise to WT F_2_ plants, 11 seeds from the red sector of plant#1 and 11 seeds from the orange part of the fruit were sown for comparison. Four IHSR WT, and one IHSR mutant progeny were found from the 11 red WT sector progeny. Two more IHSR WT progeny were found from the 11 tangerine fruit part progeny. In total, 7 IHSR progeny out of 22 F_2_ plants were obtained from red sector fruit#1 of plant#1. In F_1_ 8–9 DSB target plant#2 fruit#1, the red sector was present in the pericarp only, therefore, seeds were collected from the fruit regardless of the sector position. Twenty-two seeds were grown. Three IHSR WT and one IHSR mutant progeny were found out of 22 F_2_ progeny.

**Figure 5:**
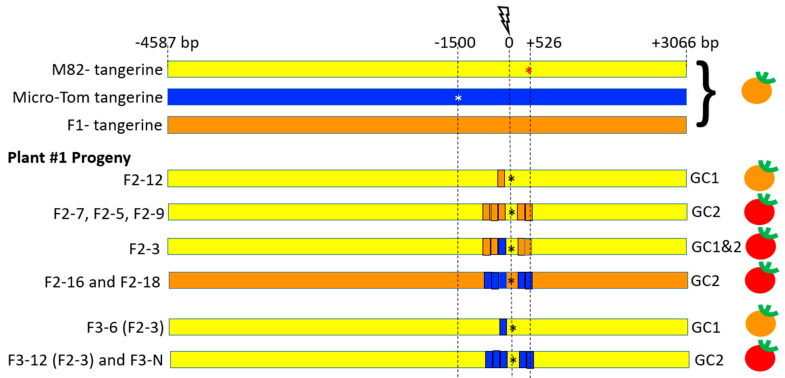
Schematic representation of IHSR events in the *CRTISO* 8–9 region around the targeted DSB in plant #1. The lightning bolt represents the CRISPR/Cas9 DSB site in the intron between exons 8 and 9 of the *CRTISO* locus. The DSB was induced between the two mutations in the *tangerine* alleles in the backgrounds of M82 (red star, 1500 bp upstream from the DSB) and Micro-Tom (white star, 526 bp downstream of the break). The F_1_ has also a tangerine phenotype, unless an IHSR event occurs. In progeny of plant #1 where a red fruit sector was observed, two types of gene conversion events were observed. GC1′s conversion tract did not span any of the two mutations and had a tangerine phenotype. For GC2, the conversion tract enabled to correct the close-by M82 mutation in a M82 chromosome background, giving rise to a dominant red fruit phenotype. SNPs that enabled the monitoring of the origin of the parental genotype are shown in yellow for M82 homozygotes, blue for Micro-Tom homozygotes, and orange for the heterozygote. Self-pollinated F_3_ plants were grown to obtain a homozygous conversion tract and to confirm the sequence of these tracts and the plant phenotype. F_3_-N refers to several F_3_ progeny that had the same genotype. The black star represents NHEJ DSB repair footprints that occurred in the intron, probably following recombination. The fruit color phenotype is indicated on the right side. A full detail of the various alleles’ sequences in all progeny is shown in Supplementary Figure S2.

**Figure 6:**
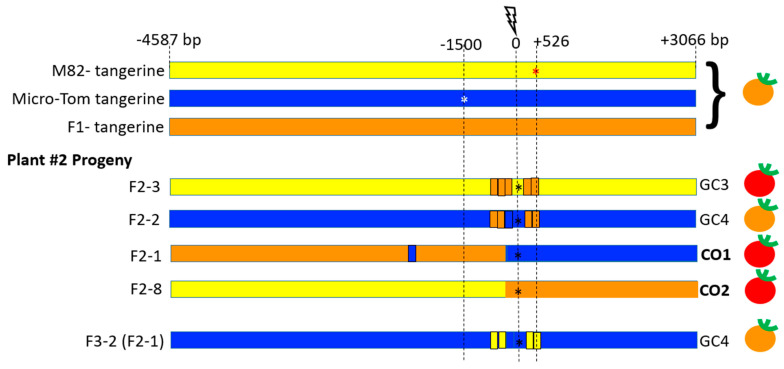
Schematic representation of IHSR events in the *CRTISO* 8–9 region around the targeted DSB in plant #2. The lightning bolt represents the CRISPR/Cas9 DSB site in the intron between exons 8 and 9 of the *CRTISO* locus. The DSB was induced between the two mutations in the *tangerine* alleles in the backgrounds of M82 (red star, 1500 bp upstream from the DSB) and Micro-Tom (white star, 526 bp downstream of the break). The F_1_ has also a tangerine phenotype, unless an IHSR event occurs. In progeny of plant #2 where a red fruit sector was observed, two types of gene conversion events were observed as well as two putative crossover events. The GC3 conversion tract was similar in sequence to GC2 in plant #1 and gave rise to a red fruit. GC4 did not restore gene activity, even though it corrected the M82 mutation but it was homozygous for the Micro-Tom mutation. The two putative crossover events both gave rise to red fruits; CO1 had a conversion tract and CO2 did not show evidence for a conversion tract. The fruit color phenotype is indicated on the right side. All plants showed NHEJ DSB repair footprints that occurred in the intron, probably following recombination (black star). A full detail of the various alleles’ sequences in all progeny is shown in Supplementary Figure S3.

We next characterized IHSR events in target 8–9 plant#1 fruit#1 (Figure 5), and plant#2 fruit#1 (Figure 6), F_2_ and F_3_ progeny by Sanger sequencing around the DSB site. The sequence data for this analysis (detailed in Supplementary Figures S2 and S3 and shown schematically in Figure 5 and Figure 6) provided insight into conversion tract length and continuity and suggested which repair pathway occurred, namely GC or CO. F_3_ plants homozygous in the *CRTISO* region were selected to facilitate sequence analysis and further confirm germinal transmission of the IHSR events. Target 8–9 from F_1_ plant#1 fruit#1 gave seven F_2_ progeny with IHSR events out of 22 progeny: five F_2_ IHSR progeny were derived from the red sector (Figure 5, Supplementary Figure S2): Plants F_2_-7, F_2_-3, F_2_-5, F_2_-9 which bear red fruits (WT), and plant F_2_-12 bearing tangerine fruits. Two F_2_ IHSR progeny derived from the tangerine sector of F_1_ plant#1, F_2_-16 and F_2_-18 bear red fruits (WT). All F_2_ and F_3_ plants contained SpCas9. Among this group, two independent types of gene conversion events, GC1 and GC2, were found. In GC1, the DSB was repaired by HR that led to copying a segment of ~50 bp length from the MT homologue to the targeted M82 allele, which generated a *tangerine CRTISO* allele (Figure 5, Supplementary Figure S2, Plant F_2_-12, and F_3_-6 (F_2_-3)). In GC2, the DSB was repaired by HR that led to copying a segment of ~983 bp from the MT homologue to the targeted M82 allele, resulting in the repair of the M82 mutation and generation of a WT *CRTISO* allele. The GC2 conversion tract is continuous and the DSB site is located in the middle of it between coordinates −456 and +526 (Figure 5, plants F_2_-7, F_2_-5, F_2_-9, F_2_-3, F2-16, F_2_-18; F_3_-12(F_2_-3), F_3_-N).

**Figure 7:**
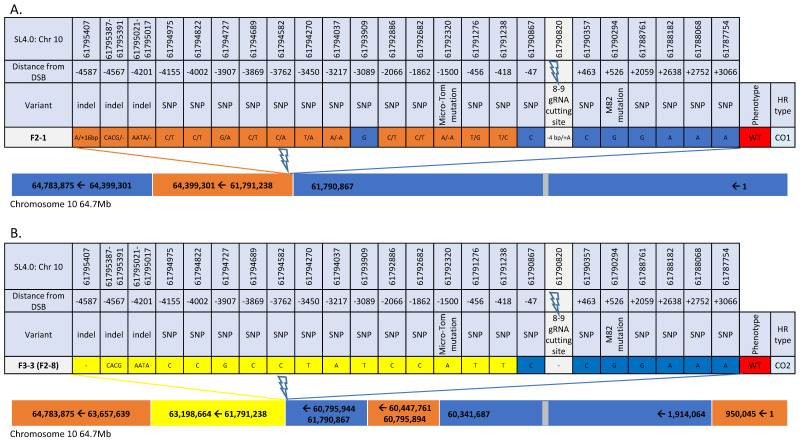
Whole genome sequencing of the two CO events from Plant#2 DSB-induced in *CRTISO* 8–9. Each plant was sent for whole genome sequencing. (**A**) F_2_-1: F_2_ plant 1 and (**B**) F_3_-3 (F_2_-8) F_3_ plant 3, progeny of F_2_ plant 8 homozygote in the *CRTISO* region. Transition from one parental type, M82 (yellow) and Micro-Tom (blue), to a heterozygote state (orange), or to the other parental type correspond to IHSR events. Indels at the DSB site represent NHEJ events in one or both alleles. DSB site footprint was determined to be homozygote (single character), heterozygote (two characters divided by “/”), “-” in case no footprint is found. Lightning bolts represent the CRISPR/Cas9 DSB region. Grey boxes represents a rough estimate of the centromere region (SL4.0 Chr10: 22482995) based on [23].

Of the 22 F_2_ progeny from F_1_-plant# 2, fruit#1 showing IHSR events, 4 were identified from target 8–9 (Figure 6, Supplementary Figure S3). Plant F_2_-2 bore tangerine fruits, and plants F_2_-3, F_2_-1, and F_2_-8 bore red fruits. All F_2_ and F_3_ plants contained SpCas9 except for plant F_2_-8 (F_1_-2) and its progeny. Among this group, two independent types of IHSR events were found, GC3 and GC4, and two independent putative CO events. GC3 and GC4 IHSR repair events both appear to result from a DSB in F_1_ parent plant (red sector in the fruit, Figure 4B). GC3 tract from F_1_ plant#2 is similar in length and coordinates/location to GC2 in F_1_ plant#1. However, it is clear that these are two independent events because each occurred in a different plant and because we see that they have a different NHEJ footprint. In GC4, the DSB was repaired by HR leading to copying a segment of ~983 bp from the M82 homologue to the targeted MT allele, resulting in both MT and M82 mutations in cis, and generation of double mutant *tangerine CRTISO* allele. The GC4 conversion tract is interrupted with the DSB site located in the center. CO1 and CO2 are putative crossover events as determined by Sanger sequencing around the DSB region (Figure 6, Supplementary Figure S3). They were further analyzed by whole genome sequencing (WGS) to confirm the presence of COs rather than long conversion tracts. WGS of plant F_2_-1 confirmed that an induced crossover event, CO1, had occurred, taking place precisely at the DSB-induced site and consisting of a very large segment (Figure 7A). CO1 has an interrupted conversion tract and a NHEJ footprint (Figure 7A). Note that plant F_2_-1 had no seeds. WGS of CO2 also confirmed that it corresponds to an induced CO event. For CO2, we sequenced the F_3_ progeny (Plant F_3_-3 (F_2_-8)) that was homozygous around the CO site. CO2 showed a clean transition (from the centromere direction to the telomere) from MT SNPs to M82 SNPs and has no NHEJ footprint at the target 8–9 break site (Figure 7B). Both CO1 and CO2 events gave rise to a WT *CRTISO* allele. Additionally, they both have a CO junction that occurred between 47 bp and 418 bp from the DSB site towards the telomere direction. The transition between the genotypes in both events continues in each direction at least 1 Mb from the 8–9 DSB. Further CO events are also detected and are likely products of meiotic CO events unrelated to the DSB in target 8–9.

## 4. Discussion

In a previous study, we showed that somatic homologous recombination between homologs can be targeted via DSB induction at the tomato *Phytoene Synthase1* locus [10]. We extended this work by (i) testing a new locus (*CRTISO*), (ii) developing a new gain-of-function phenotypic IHSR assay targeting introns (the previous assay was loss of function), (iii) expanding the number of events analyzed, (iv) demonstrating the occurrence of two targeted COs through whole genome sequencing (the single earlier case was a putative CO event). We confirm earlier findings that IHSR events can be germinally transmitted and that gene conversion tracts could be continuous or interrupted.

The red sector fruit phenotypic marker was a reliable marker to detect germinally transmitted IHSR events, but the presence of red sectors was not a guarantee of germinal transmission, in particular with small-size sectors where there is only partial correspondence between the maternal fruit tissue and the gametes. Nevertheless, the correlation between the presence of red sectors in fruit somatic tissues of F_1_ plants and the finding of recombination between the two very close (2 Kb apart) mutations in *CRTISO* in the progenies supports the somatic origin of these recombination events. Moreover, a meiotic origin for such events is very unlikely because of the proximity of the two mutations, because of the presence of red sectors in F_1_ plants and because of the lack of red sectors in all F_2_ negative control plants.

Out of eighteen transgenic F_1_ plants, we had two plants that together gave six germinally transmitted somatic recombinant alleles. Of these six alleles, five were different. Three alleles were GC events, and two were CO events. Three GC alleles and one CO event were successfully transmitted to further F_3_ fertile plants. By analyzing the conversion tract, we found that two independent fruits of two independent plants had the same NCO recombinant allele, with a continuous conversion tract of ~983 bp length (Figure 5 and Figure 6, GC2). This could be due to a preferential IHSR repair or to our limited ability to determine size precisely due to low polymorphism between the two parental alleles.

One out of three independent GC tracts as well as one out of two CO conversion tracts were interrupted. This phenomenon was observed in yeast [24] and in tomatoes [10].

In F_2_ plant#1, we observed two types of GC events, with ~50 bp (GC1) and ~1000 bp (GC2) conversion tract lengths, both of which were due to invasion of the M82 allele into the MT allele. These could hint to a Synthesis Dependent Strand Annealing (SDSA) mechanism leading to GC [25]. In F_2_ plant#2, we detected two types of GC events, with ~1000 bp tract lengths (GC3, GC4) spanning the same SNPs. GC3 is due to invasion of the M82 allele into the MT allele, generating a WT allele. GC4 is due to invasion of the MT allele into the M82, resulting in formation of an allele with both mutations.

Intriguingly, having a strong DSB induction was not sufficient to induce IHSR. Indeed, only one out of three gRNAs tested showed high DSB-inducing activity that yielded red sectors indicative of IHSR. The factors determining the ability of a given sequence to be repaired by IHSR remains unclear. Is it due to the “loop-out” of some sequences, the kinetics of repair at this locus, chromatin features, or somatic association? Our sample remains too small to determine the underlying mechanism.

The novel assay that we designed and described in this work could be used for future research such as the characterization of candidate genes involved in DNA DSB repair and their effect in IHSR, and the rates of crossover versus non-crossover. In addition, a better understanding of the IHSR phenomenon might be useful to develop precise genome editing breeding tools, for both vegetatively propagated crops and sexually propagated crops, by inducing homologous recombination in somatic cells and thus bypassing the need for meiotic recombination and its limitations.

## Data Availability

The data presented in this study are available on request from the corresponding author.

## Supplementary Materials

The following are available online at https://www.mdpi.com/2073-4425/12/1/59/s1, Supplementary Table S1. Primers used for T-DNA positive plants screening and selection, Supplementary Table S2. Primers used for sequencing of the *CRTISO* parental mutant lines Micro-Tom 18-3, M82 e3406, and their F_1_ hybrid plants, Supplementary Table S3. SpCas9 DSB targets design and considerations, Supplementary Table S4. Primers for Illumina high throughput sequencing of DSB targets NHEJ footprints, Supplementary Table S5. Primers used for sequencing of *CRTISO* region in F_2_ and F_3_ plants, Supplementary Table S6. *CRTISO* gene coordinates of targeted DSB IHSR assay, Supplementary Table S7. *CRTISO* targeted DSB IHSR phenotypic assay targets comparison, Supplementary Figure S1. NHEJ frequency in F_1_ plants of *CRTISO* assay DSB targets, Supplementary Figure S2. Sequencing of IHSR events in the *CRTISO* 8–9 region around the targeted DSB in plant#1, Supplementary Figure S3. Sequencing of IHSR events in the *CRTISO* 8–9 region around the targeted DSB in plant#2.
