# Supplementary material for: CRISPR/Cas9 Induced Somatic Recombination at the CRTISO Locus in Tomato
Source: Genes (Basel). 2020 Dec 31;12(1):59. doi: 10.3390/genes12010059 (PMC7824628; doi:10.3390/genes12010059)
Supplement: Supplementary file 1 [file genes-12-00059-s001.pdf]

CRISPR/Cas9 induced somatic recombination at the *CRTISO* locus in tomato

Authors: Ilan Ben Shlush[1*], Aviva Samach[1*], Cathy Melamed-Bessudo[1], Daniela Ben-Tov[1], Tal Dahan-Meir[1], Shdema Filler-Hayut[1], Avraham A. Levy[1]

[1] **Institution:** Department of Plant and Environmental Sciences, The Weizmann Institute of Science, Rehovot, Israel.

* Equal contributors

Corresponding Author: Avraham A. Levy - Department of Plant and Environmental Sciences, The Weizmann Institute of Science, Rehovot 7610001, Israel. Telephone: (W) 972 8 934 2734. Fax: 972 8 934 4181

E-mail: avi.levy@weizmann.ac.il

SUPPLEMENTARY MATERIALS

Primer name	Sequence
Cas9 long_F	CAGAATGAGAAGCTCTACCTCTACTACCTC
Cas9 long_R	GAAATTCATGATGTTAGAGTAGAAGAAATACTTAG

Supplementary Table S1. Primers used for T-DNA positive plants screening and selection. SpCas9 primers amplifying Cas9 sequence from the T-DNA plasmid for transgenic plants T-DNA presence verification. The amplicon size is 711 bp.

Region amplified	Primer name	Sequence
Upstream to Exon 1	**CRTISO_0_F**	ACACCCTTTTGCCACTTCAC
	crtiso_donor_5end_R	CCAAACACCTAGTGAAAAGC
	**CRTISO_0_R**	GCTTGCTGGCTTTGGTTAAT
Upstream to Exon 1 to Exon 4	**CRTISO_1_F**	TCTGAATTCACCTCCTCACG
	**CRTISO_3_R**	AGACAGCAACCCAGGATCTC
Exon 4 to Exon 11 (Both mutations)	**CRTISO_4_F**	TCTTTCACGCTGATGTGTGC
	**CRTISO_6_R**	GGAAGCAACTATCGCCAAC
Exon 12 to 13	**CRTISO_7_F**	GGGAATGCCTTTCAATACCACTG
	CRTISO_7.5_R	GCTCGACGTTGTAAATACTC
	**CRTISO_7_R**	CCTTTGGCAGAAAGTTGCAGA
Downstream to Exon 13	**CRTISO_8_F**	CAGATGTGCTGGACAGTGC
	**CRTISO_8_R**	GAACCTGTAGCCTGAATGG

Supplementary Table S2. Primers used for sequencing of the *crtiso* parental mutant lines Micro-Tom 18-3, M82 e3406, and their F_1 hybrid plants. We used PCR amplification and Sanger sequencing to detect SNPs that can serve as markers for HR. Primer pairs marked in bold were used for the PCR amplification of genomic DNA fragments. All primers were used for Sanger sequencing. Primers CRTISO_4_F and CRTISO_6_R were used for the verification of Micro-Tom 18-3 -A mutation on Exon 4 and M82 e3406 G->A mutation on Exon 11 in the parental lines and in F_1 hybrid plants.

Target	Target sequence (PAM in lower case)	SL4.0 chromosome 10 coordinate	Strand	Distance from MT mutation [bp]	Distance from M82 mutation [bp]	Distance from 5' GU AS site [bp]	Distance from 3' AG AS site [bp]
CRTISO_6-7 gRNA	TCTTTGCCAGTATCTGCGCAagg	61791282	+	1039	989	594	34
CRTISO_7-8 gRNA	GTGCAAGAATACCACAGTACtgg	61791100	-	1221	806	65	70
CRTISO_8-9 gRNA	TAAAATCAAGGAATCATGAAtgg	61790820	-	1500	527	56	18
CRTISO_9-10 gRNA	AAGAGACATAGATGTGGAAGagg	61790622	-	1699	328	42	60

Supplementary Table S3. SpCas9 DSB targets design and considerations.

Four SpCas9 gRNAs were designed to induce DSBs within introns in between *crtiso* MT and M82 mutations in F_1 plants. Targets sequence including PAM (in lower case) and direction (strand) are shown.

Primer name	Sequence	Size [bp]
**6-7-8_F**	GTGTAGCATCTTCAATGTTGTCT	278
**6-7-8_R new**	ACACTTGACATGAGATGACGAGA	
**7-8-9_F**	CTTTCGTCTTAGGGCCAGTACT	341
**7-8-9_R**	TCTCCAAATTTGTCCAATCATCC	
**8-9-10_F new**	TGTCACCATTTTGTCCTCGAG	328
**8-9-10_R**	AAGGGCCATACTTTAATTTCCAT	

Supplementary Table S4. Primers for Illumina high throughput sequencing of DSB targets NHEJ footprints. We designed three amplicons, each one had amplified two targets. Both 7-8 and 8-9 targets were amplified twice.

Region amplified	Primer name	Sequence
CRTISO_0_F to CRTISO_1R : 1736 bp	CRTISO_0_F	ACACCCTTTTGCCACTTCAC
	CRTISO_1R	ACTGCCATAGCTCTCCACTC
CRTISO_3_F to CRTISO_3_R : 937 bp	CRTISO_3_F	ACTGCCATAGCTCTCCACTC
	CRTISO_3_R	AGACAGCAACCCAGGATCTC
CRTISO_4_F to CRTISO_5_R : 1755 bp	CRTISO_4_F	TCTTTCACGCTGATGTGTGC
	CRTISO_5_R	CCCAATCTTCAATGCTCGAT
8-9-10_F_new to CRTISO_6_R : 845 bp	8-9-10_F_new	TGTCACCATTTTGTCCTCGAG
	CRTISO_6_R	GGAAGCAACTATCGCCAAC
CRTISO_11_F to CRTISO_11_R : 1422bp	CRTISO_11_F	CCAAACAGAGGTGACTTCAAAGA
	CRTISO_11_R	GTGCATGGTTAAACATAGGAACATGA

Supplementary Table S5. Primers used for sequencing of *CRTISO* region in F_2 and F_3 plants. We used PCR amplification and Sanger sequencing to detect SNPs that can serve as markers for IHSR events. Primer pairs were used for the PCR amplification of genomic DNA fragments, and Sanger sequencing.

SL4.0: Chr 10	Variant	M82 *crtiso*	Micro-Tom *crtiso*
61795407	indel	-	+16bp
61795387-61795391	indel	CACG	-
61795021-61795017	indel	AATA	-
61794975	SNP	C	T
61794822	SNP	C	T
61794727	SNP	G	A
61794689	SNP	C	T
61794582	SNP	C	A
61794270	SNP	T	A
61794037	SNP	A	-A
61793909	SNP	T	G
61792886	SNP	C	T
61792682	SNP	C	T
61792320	Micro-Tom mutation	A	-A
61791282	6-7 gRNA cutting site	-	+
61791276	SNP	T	G
61791238	SNP	T	C
61791100	7-8 gRNA cutting site	+	+
61790867	SNP	G	C
61790820	8-9 gRNA cutting site	+	+
61790622	9-10 gRNA cutting site	+	+
61790357	SNP	T	C
61790294	M82 mutation	A	G
61788761	SNP	C	G
61788182	SNP	G	A
61788068	SNP	G	A
61787754	SNP	G	A

Supplementary Table S6. *CRTISO* gene coordinates of targeted DSB IHSR assay. The SNPs, mutations and DSB sites SL4.0 location and order are presented in the gene table for both M82 and Micro-Tom parental mutant alleles.

	M82 *crtiso* (no repair template)					F1 M82/ Micro-Tom *crtiso*				
**DSB Target**	**Cas9 only**	**6-7**	**7-8**	**8-9**	**9-10**	**Cas9 only**	**6-7**	**7-8**	**8-9**	**9-10**
**Confirmed NHEJ Cas9 activity**	No	Yes	Yes	Yes	No	No	Yes	Yes	Yes	No
**F_1 plants**	4	3	8	6	7	9	21	10	18	10
**F_1 plants with red sector fruits**	0	0	0	0	0	0	0	0	5	0
**F_1 red sector fruits**	0	0	0	0	0	0	0	0	10	0
**F_1 red sector fruits with germinally transmitted IHSR**	0	0	0	0	0	0	0	0	2	0

Supplementary Table S7. *CRTISO* targeted DSB IHSR phenotypic assay targets comparison. F_1 plants with the four targets, and control plants are presented. We used two types of controls: F_1 with Cas9 only but no gRNA (Cas9 only) – to show that gRNA is mandatory for DSB induction; M82 plants with each of the four targets – to show that we do not detect WT phenotype due to IHSR with the same repair template, but only when we have both M82 and Micro-Tom *tangerine* in the F_1. All the controls did not have any WT phenotype. Red sector fruits indicating late IHSR event, were detected in CRTISO DSB target 8-9 only. Number of red sector fruits and number of WT F_2 plants originated from them, are presented to evaluate germinal transition rate of IHSR events. Three tangerine fruits from each plant were collected and tested out of five plants of each target. It served as control to test whether WT F_2 plants can originate from tangerine fruits. DSB target 8-9 had red sector fruits originating from five plants, with varying numbers of one to three per plant, and ten red sector fruits in total. One fruit was sterile and four other fruits had only 1- 3 seeds. The other five red sector fruits had 13-22 seeds.

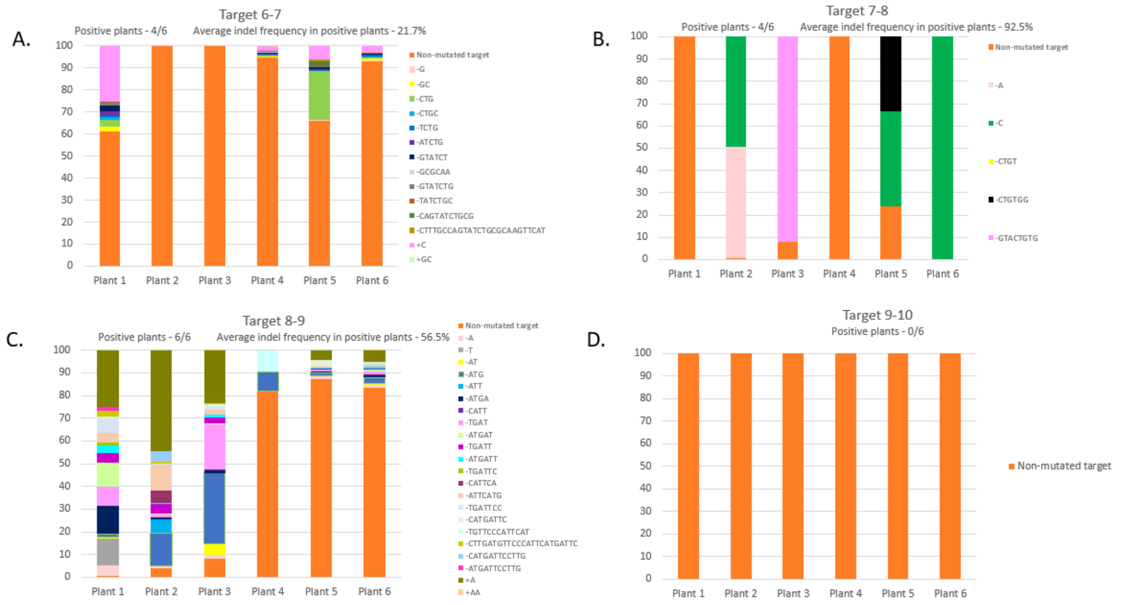

Supplementary Figure S1. NHEJ frequency in F_1 plants of *CRTISO* assay DSB targets. Illumina NextSeq high-throughput sequencing results for F_1 plants analyzed using NGS Cas-Analyzer and presented for all four *CRTISO* assay DSB targets (A-D). Each panel represents one of the four targets. For each target six F_1 plants were analyzed. The indels frequency was calculated by the number of indel reads out of the total number of reads (including non-mutated target footprint) and shown as a percentage. No indels were detected in three control plants with Cas9 only.

	SL4.0: Chr 10	61795407	61795387-61795391	61795021-61795017	61794975	61794822	61794727	61794689	61794582	61794270	61794037	61793909	61792886	61792682	61792320	61791276	61791238	61790867	61790820	61790357	61790294	61788761	61788182	61788068	61787754		
	Distance from DSB	-4587	-4567	-4201	-4155	-4002	-3907	-3869	-3762	-3450	-3217	-3089	-2066	-1862	-1500	-456	-418	-47		+463	+526	+2059	+2638	+2752	+3066		
	Variant	indel	indel	indel	SNP	SNP	SNP	SNP	SNP	SNP	SNP	SNP	SNP	SNP	Micro-Tom mutation	SNP	SNP	SNP	8-9 gRNA cutting site	SNP	M82 mutation	SNP	SNP	SNP	SNP	Phenotype	HR type
	**M82 tangerine**	A	CACG	AATA	C	C	G	C	C	T	A	T	C	C	A	T	T	G	-	T	A	C	G	G	G	tangerine	-
	**Micro-Tom tangerine**	+16bp	-	-	T	T	A	T	A	A	-A	G	T	T	-A	G	C	C	-	C	G	G	A	A	A	tangerine	-
	**F1 tangerine**	A/+16bp	CACG/-	AATA/-	C/T	C/T	G/A	C/T	C/A	T/A	A/-A	T/G	C/T	C/T	A/-A	T/G	T/C	G/C	-	T/C	A/G	C/G	G/A	G/A	G/A	tangerine	-
red sector	**F2-12**	A	CACG	AATA	C	C	G	C	C	T	A	T	C	C	A	T	T	G/C	-4bp / +A	T	A	C	G	G	G	tangerine	GC1
red sector	**F2-7**	A	CACG	AATA	C	C	G	C	C	T	A	T	C	C	A	T/G	T/C	G/C	-5bp / +A	T/C	A/G	C	G	G	G	WT	GC2
red sector	**F2-3**	A	CACG	AATA	C	C	G	C	C	T	A	T	C	C	A	T/G	T/C	C	-4bp / +A	T/C	A/G	C	G	G	G	WT	GC1, GC2
red sector	**F2-5**	A	CACG	AATA	C	C	G	C	C	T	A	T	C	C	A	T/G	T/C	G/C	-7bp / +A	T/C	A/G	C	G	G	G	WT	GC2
red sector	**F2-9**	A	CACG	AATA	C	C	G	C	C	T	A	T	C	C	A	T/G	T/C	G/C	+A	T/C	A/G	C	G	G	G	WT	GC2
tangerine sector	**F2-16**	A/+16bp	CACG/-	AATA/----	C/T	C/T	G/A	C/T	C/A	T/A	A/-A	T/G	C/T	C/T	A/-A	G	C	C	+A	C	G	C/G	G/A	G/A	G/A	WT	GC2
tangerine sector	**F2-18**	A/+16bp	CACG/-	AATA/----	C/T	C/T	G/A	C/T	C/A	T/A	A/-A	T/G	C/T	C/T	A/-A	G	C	C	+A	C	G	C/G	G/A	G/A	G/A	WT	GC2
	**F2-12**	A	CACG	AATA	C	C	G	C	C	T	A	T	C	C	A	T	T	C	-4bp	T	A	C	G	G	G	tangerine	GC1
	**F2-7**	A	CACG	AATA	C	C	G	C	C	T	A	T	C	C	A	G	C	C	+A	C	G	C	G	G	G	WT	GC2
	**F3-6 (F2-3)**	A	CACG	AATA	C	C	G	C	C	T	A	T	C	C	A	T	T	C	-4bp	T	A	C	G	G	G	tangerine	GC1
	**F3-12 (F2-3)**	A	CACG	AATA	C	C	G	C	C	T	A	T	C	C	A	G	C	C	+A	C	G	C	G	G	G	WT	GC2
	**F3-1 (F2-5)**	A	CACG	AATA	C	C	G	C	C	T	A	T	C	C	A	G	C	C	+A	C	G	C	G	G	G	WT	GC2
	**F3-2 (F2-9)**	A	CACG	AATA	C	C	G	C	C	T	A	T	C	C	A	G	C	C	+A	C	G	C	G	G	G	WT	GC2
	**F3-3 (F2-16)**	A	CACG	AATA	C	C	G	C	C	T	A	T	C	C	A	G	C	C	+A	C	G	C	G	G	G	WT	GC2
	**F3-2 (F2-18)**	A	CACG	AATA	C	C	G	C	C	T	A	T	C	C	A	G	C	C	+A	C	G	C	G	G	G	WT	GC2

Supplementary Figure S2. Sequencing of IHSR events in the *CRTISO* 8-9 region around the targeted DSB in plant#1. SL4.0 chromosome 10 61795407 to 61787754, spanning the *tangerine* mutations of both Micro-Tom and M82, were sequenced for each plant by Sanger sequencing. Lightning bolt represents the CRISPR/Cas9 DSB region. Sequencing of PCR products in F_2 plants is shown on the left side as light grey. F_2 plant single molecule sequencing is shown as dark grey highlight (See HR events analysis in the *CRTISO* region in the methods section). F_3 homozygote plants are shown as black highlight. F_2-3, means F_2 plant number 3. F_3-6 (F_2-3), means F_3 plant 6, progeny of F_2 plant 3. Transition from one parental type, M82 (yellow) and Micro-Tom (blue), to a heterozygote state (orange), or to the other parental type correspond to IHSR events (GC). Indels at the DSB site correspond to NHEJ events in one or both alleles. DSB site footprint was determined to be homozygote (single character), heterozygote (two characters divided by "/"). The first five F_2 plants, originated from the red sector and the subsequent two F_2 plants originated from the tangerine part of the fruit. Overall, two

independent types of GC events were found, GC1 and GC2 (see right column).

SL4.0: Chr 10	61795407	61795387-61795391	61795021-61795017	61794975	61794822	61794727	61794689	61794582	61794270	61794037	61793909	61792886	61792682	61792320	61791276	61791238	61790867	61790820	61790357	61790294	61788761	61788182	61788068	61787754		
Distance from DSB	-4587	-4567	-4201	-4155	-4002	-3907	-3869	-3762	-3450	-3217	-3089	-2066	-1862	-1500	-456	-418	-47		+463	+526	+2059	+2638	+2752	+3066		
Variant	indel	indel	indel	SNP	SNP	SNP	SNP	SNP	SNP	SNP	SNP	SNP	SNP	Micro-Tom mutation	SNP	SNP	SNP	8-9 gRNA cutting site	SNP	M82 mutation	SNP	SNP	SNP	SNP	Phenotype	HR type
M82 tangerine	-	CACG	AATA	C	C	G	C	C	T	A	T	C	C	A	T	T	G	-	T	A	C	G	G	G	tangerine	-
Micro-Tom tangerine	+16bp	-	-	T	T	A	T	A	A	-A	G	T	T	-A	G	C	C	-	C	G	G	A	A	A	Tangerine	-
F1 tangerine	A/+16bp	CACG/-	AATA/-	C/T	C/T	G/A	C/T	C/A	T/A	A/-A	T/G	C/T	C/T	A/-A	T/G	T/C	G/C	-	T/C	A/G	C/G	G/A	G/A	G/A	tangerine	-
F2-3	-	CACG	AATA	C	C	G	C	C	T	A	T	C	C	A	T/G	T/C	G/C	-3 bp/+A	T/C	A/G	C	G	G	G	WT	GC3
F2-2	+16bp	-	-	T	T	A	T	A	A	-A	G	T	T	-A	T/G	T/C	C	-1/-2/-3/-4 bp	T/C	A/G	G	A	A	A	tangerine	GC4
F2-1	A/+16bp	CACG/-	AATA/-	C/T	C/T	G/A	C/T	C/A	T/A	A/-A	G	C/T	C/T	A/-A	T/G	T/C	C	-4 bp/+A	C	G	G	A	A	A	WT	CO1
F2-8	-	CACG	AATA	C	C	G	C	C	T	A	T	C	C	A	T	T	G/C	-/+A	T/C	A/G	G/C	G/A	G/A	G/A	WT	CO2
F2-3	-	CACG	AATA	C	C	G	C	C	T	A	T	C	C	A	G	C	C	+A	C	G	C	G	G	G	tangerine	GC3
F3-2 (F2-1)	+16bp	-	-	T	T	A	T	A	A	-A	G	T	T	-A	T	T	C	+A	T	A	G	A	A	A	tangerine	GC4

Supplementary Figure S3. Sequencing of IHSR events in the *CRTISO* 8-9 region around the targeted DSB in plant#2. SL4.0 chromosome 10 61795407 to 61787754, spanning the *tangerine* mutations of both Micro-Tom and M82, were sequenced for each plant by Sanger sequencing. Lightning bolt represents the CRISPR/Cas9 DSB region. Sequencing of PCR products in F_2 plants is shown on the left side as light grey. F_2 plant single molecule sequencing is shown as dark grey highlight (See HR events analysis in the *CRTISO* region in the methods section). F_3 homozygote plants sequence is shown as black highlight. F_2-1, means F_2 plant number 1. F_3-2 (F_2-1), means F_3 plant 2, progeny of F_2 plant 1. Transition from one parental type, M82 (yellow) and Micro-Tom (blue), to a heterozygote state (orange), or to the other parental type correspond to IHSR events (GC). Indels at the DSB site represent NHEJ events in one or both alleles. DSB site footprint was determined to be homozygote (single character), heterozygote (two characters divided by "/"), "-" in case no footprint is found. Four F_2 IHSR events were verified: 2 (tangerine), and 3, 1, 8 (WT). Among this group, two independent types of GC events were found, GC2 and GC3. GC3 is an interrupted conversion tract event. Additionally, two CO events were found in this group, and verified by WGS.
